# Alteration of long non-coding RNAs and mRNAs expression profiles by compound heterozygous ASXL3 mutations in the mouse brain

**DOI:** 10.1080/21655979.2021.1974811

**Published:** 2021-09-24

**Authors:** Songhui Zhang, Fang Fu, Li Zhen, Ru Li, Can Liao

**Affiliations:** a The Second School of Clinical Medicine, Southern Medical University, Guangzhou, China; b Department of Obstetrics, The Second Affiliated Hospital, Guangzhou Medical University, Guangzhou, China; c Prenatal Diagnostic Center, Guangzhou Women and Children’s Medical Center, Guangzhou, China

**Keywords:** Long non-coding RNA profile, messenger RNA profile, additional sex combs-like 3, cerebrum, cerebellum

## Abstract

Compound mutations in the additional sex combs-like 3 (ASXL3) gene greatly impact the expression of long non-coding RNAs (lncRNAs) and messenger RNAs (mRNAs) in mouse myocardial tissues. Little is known about ASXL3 mutation effects on lncRNAs and mRNAs expression in the cerebrum and cerebellum. This study aims to clarify this point using quantitative real-time polymerase chain reaction and Western blotting. Transcriptome analysis based on RNA-seq followed by bioinformatics analysis were used to compare lncRNA and mRNA expression profiles. Cell proliferation, cell cycle progression, and apoptosis were evaluated after silencing of ASXL3 expression using the 3-(4,5-dimethylthiazol-2-yl)-5-(3-carboxymethoxyphenyl)-2-(4- sulfophenyl)-2 H-tetrazolium method and flow cytometry. Results showed that ASXL3 gene expression was decreased in the cerebrum and cerebellum of mice with ASXL3 P723R*P1817A mutations. We identified 319 lncRNAs and 252 mRNAs differentially expressed in the cerebrum of ASXL3 P723R*P1817A mutant mice. In the cerebellum of ASXL3 P723R*P1817A mutant mice, 5330 lncRNAs and 2204 mRNAs were differentially expressed. Differentially expressed lncRNAs and mRNAs were widely distributed across the mouse genome and were associated with various biological processes and pathways. ASXL3 silencing by siRNA transfection affected the proliferation, cell cycle progression, and apoptosis of neural cells. Therefore, the ASXL3 P723R*P1817A mutations greatly modify the lncRNA and mRNA expression profiles in the mouse cerebrum and cerebellum.

## 1. Introduction

The cerebrum, which contains the cerebral cortex and subcortical structures, and the cerebellum are two major components of the mammalian central nervous system, responsible for the control of all the body voluntary actions and most cognitive functions, including language, attention, and fear or pleasure responses [1,2]. The development and the physiological functions of neurons and glial cells in the cerebrum and cerebellum are precisely controlled by complex networks of signal transduction and gene expression [3,4]. The deregulation of the functional expression of genes, especially those associated with major signaling cascades, in neurons and glial cells has also been involved in the pathogenic processes of various severe disorders of the human brain such as glioma [5,6]. However, the mechanisms controlling the gene expression in neuronal and glial cells and associated with development, functioning, and pathogenesis of the human cerebrum and cerebellum are remain poorly understood.

Long non-coding RNAs (lncRNAs) are transcripts containing more than 200 nucleotides, which are associated with various biological processes and disease pathogenesis [7]. The synthesis of lncRNAs is carried out by RNA Polymerase II-mediated genome transcription and can be followed by 5′ capping, alternative splicing, and polyadenylation, similarly to the mechanisms involved in the transcription and processing of protein coding messenger RNAs (mRNAs) [8]. Additionally, lncRNAs can be encoded by genomic sequences overlapping with protein-coding genes or intergenic sequences. The different genomic locations and orientations of lncRNAs might influence their mechanism of action [9]. Currently, some lncRNAs are known to modulate the transcription of other genes by forming ribonucleoprotein complexes together with chromatin-regulating proteins [10]. For instance, Xist, a well-studied lncRNA, was reported to silence the gene transcription in one X chromosome by interacting with different proteins, such as SPEN, heterogeneous nuclear ribonucleoprotein U family proteins, and CDKN1A interacting zinc finger protein 1 [11]. The potent capability of lncRNAs to influence gene expression suggests that they have a role in various biological and pathogenic processes.

Recent studies revealed that many lncRNAs are prevalently expressed in both the developing and adult central nervous systems, especially in the brain [12]. More importantly, accumulating evidence shows that an abnormal lncRNAs expression in neural tissues are closely associated with the initiation and progression of various severe neurological diseases such as glioma, autism, Alzheimer’s disease, Parkinson’s disease, and schizophrenia [13–17]. Transcriptomic analysis showed that a group of lncRNAs are acutely and dynamically expressed during neuronal activation processes [17]. Gomafu, a lncRNA associated with retinal development, directly binds several splicing factors and influences alternative splicing, being thus involved in the pathogenesis of neurological disorders including schizophrenia [17]. Recent single cell analyses based on strand-specific RNA-seq technique demonstrated that lncRNAs are expressed in the developing human brain in a cell type-specific and tissue-specific manner [18]. For instance, the lncRNA LOC646329 is highly enriched in individual radial glia cells and affects glial cell proliferation [18]. Despite the recent progress in understanding lncRNA expression and functions in the nervous system, the overall role, expression regulation, and specific mechanisms of action of lncRNAs during the cerebrum and cerebellum development and pathogenic events deserve further investigations.

The additional sex combs-like 3 (ASXL3) gene, is a protein associated with the polycomb repressive complex 2 and is upregulated in small cell lung cancer cells [19]. The patients suffering from ASLX3-related disorder rather show signs or features of autism, sleep disturbance, and feeding problem [20]. The ASXL3 gene is differentially expressed in human neural stem cells treated with dihydrotestosterone, indicating ASXL3 association with neurodevelopmental disorders including autism spectrum disorder [21]. However, the molecular events leading by ASXL3 pathogenic expression and mutation are still unclear.

Our previous investigations characterized the compound heterozygous mutations (c.2168 C > G*c.5449 C > G) of the ASXL3 gene from a Chinese family of congenital heart disease [22]. Our transcriptome study revealed significant alterations in the lncRNA and mRNA expression profiles in mouse myocardial tissues caused by the compound mutations of ASXL3 gene. Therefore, we speculated that ASXL3 compound mutations induce changes in the lncRNA and mRNA expression profiles in the nervous system. The present work aims to investigate the expression profiles of lncRNAs and mRNAs in the cerebrum and cerebellum of mice with ASXL3 mutations. We also analyzed the influence of the ASXL3 gene on neural cell functions. This study provides novel insights into the roles of the ASXL3 gene and lncRNA–mRNA interactions in brain functions and neurological disorders.

## 1. Material and Methods

### 1.1 Animals and brain tissue collection

The mutant mouse line with a heterozygous double mutation (P723R*P1817A) in the ASXL3 gene was established by the Cyagen Biosciences (Suzhou, China) as previously described [22]. Cerebrum and cerebellum tissues from double mutant and wild-type C57BL/6 mice were collected at the age of 4–6 weeks, after the mice were euthanized by anesthesia with isoflurane inhalation and instant cervical dislocation. The weights of the cerebrum and cerebellum tissues were measured separately. All experimental procedures in this study were carried out following the guideline of the European Union Directive 2010/63/EU on the protection of animals used for scientific purposes and the NIH guidelines and were approved by the Animal Care and Use Committee of the Guangzhou Medical University.

### 2.2. qRT-PCR

The relative mRNA and lncRNA expression levels in mouse tissues were evaluated by quantitative real-time polymerase chain reaction (qRT-PCR). The experimental protocol was identical to that from our previous study [22]. The sequences of the primers used in this study are listed in Table 1.

**Table 1.** Primers sequences used for quantitative real-time polymerase chain reaction

Gene Name	Primer name	Sequence (5'-3')	Product length (bp)
M-Asxl3	Forward primer	CCCTATGACCAGAACGAAGTGA	177
	Reverse primer	CCCAAAGTGTATCGTCGGGTAA	
NONMMUT130640.1	Forward primer	AGTGAGCTGGACATTCGGTG	153
	Reverse primer	TGGCACAGTTTGCAACCAAC	
NONMMUT072957.2	Forward primer	TTGCTCCACCCCAAAGAGTG	126
	Reverse primer	CTCCACCAACCCTGTGGTTT	
NONMMUT047824.2	Forward primer	TGCTGTGGCTATAACTGCGA	182
	Reverse primer	CACACATCTGCCGAGACGTA	
NONMMUT044470.2	Forward primer	GCTGAGTCAATGAGGGAAGA	288
	Reverse primer	TCCTGAGGGTTTTGGATTTT	
NONMMUT041409.2	Forward primer	TGAGTTGCCCTGAGCCATTA	113
	Reverse primer	ATCACTTGCTTGTGACCCGA	
NONMMUT135643.1	Forward primer	GTGGTCGCCTCAACCAAGTA	138
	Reverse primer	AGAACTCGCTGAGGTTGTGG	
NONMMUT021458.2	Forward primer	CGTTTTGCTCCTTGTGGGAT	141
	Reverse primer	ACAACCCACTGGTATCGGGT	
NONMMUT009850.2	Forward primer	AGCCCCTGAAAGTTGTGGTG	152
	Reverse primer	GAATCTCGTTCCCCTGCCTC	
NONMMUT008224.2	Forward primer	GCCACTGAAGGTGAGCACTA	234
	Reverse primer	TGTCTACCCCAGCTCCAATC	
NONMMUT008222.2	Forward primer	GTGCCTACCCAAGATGTGTC	112
	Reverse primer	GGAACCTCTAAGTACAGCAGCA	
NONMMUT008220.2	Forward primer	CTGTGCCTACCCAAGATGTGT	249
	Reverse primer	TCTGGCTCTGTCAAACCTGCT	
Suv39h1	Forward primer	TGACCGTTACCCTTTCGGTTT	108
	Reverse primer	ACACACTGCAACCTAGAGTCG	
Socs3	Forward primer	ATGGTCACCCACAGCAAGTTT	145
	Reverse primer	TCCAGTAGAATCCGCTCTCCT	
Slc6a4	Forward primer	TATCCAATGGGTACTCCGCAG	110
	Reverse primer	CCGTTCCCCTTGGTGAATCT	
Plod3	Forward primer	ATGTGGCTCGAACAGTTGGTG	123
	Reverse primer	TTGCCAGAATCACGTCGTAGC	
Fos	Forward primer	TACTACCATTCCCCAGCCGA	113
	Reverse primer	GCTGTCACCGTGGGGATAAA	
Ezh2	Forward primer	AGTGACTTGGATTTTCCAGCAC	100
	Reverse primer	AATTCTGTTGTAAGGGCGACC	
NONMMUT055906.2	Forward primer	TTTGGAGCCATTTGGGATGG	102
	Reverse primer	GCAGTGTGGTACGTGGCTAA	
NONMMUT058317.2	Forward primer	TTTCATGGCTCGTTCTTGCC	100
	Reverse primer	GCCTGGTGAGTTAGCCTGTC	
NONMMUT071574.2	Forward primer	CACGGGACTCAGCAGTGATA	143
	Reverse primer	GTTGACACGTTTTACGCCGA	
NONMMUT057298.2	Forward primer	AAGCAATCAAGTGGGAACCCC	244
	Reverse primer	CCACCTGGAAATGTCCCACA	
NONMMUT046493.2	Forward primer	TGCAGTTTCCCACACCCTAT	243
	Reverse primer	TCATCATGTCAGGGCCTCAA	
NONMMUT053942.2	Forward primer	GTCCTCACGTGCTGGATTCT	216
	Reverse primer	TAGCCTTGTGACTGCCAACC	
H-ASXL3	Forward primer	CTAACAATCCGCTGGTGACG	168
	Reverse primer	TGGGCGGAATTTCAACGTTT	
H-GAPDH	Forward primer	CAAATTCCATGGCACCGTCA	132
	Reverse primer	GACTCCACGACGTACTCAGC	
M-GAPDH	Forward primer	GGGTCCCAGCTTAGGTTCAT	95
	Reverse primer	TACGGCCAAATCCGTTCACA	

### 2.3 RNA sequencing and quantification

The alterations of the mRNA and lncRNA expression profiles in the cerebrum and cerebellum of wild-type and mutant mice were analyzed by RNA sequencing as described previously [22]. The lncRNAs or mRNAs were considered differentially expressed in mutant mice compared to the levels in wild-type animals for a fold change ≥ 2 and FDR < 0.001.The genomic distribution and length of differentially expressed lncRNAs and mRNAs were also analyzed separately.

### 2.4 Bioinformatics

Gene ontology (GO) enrichment analysis was carried out using the Gene Ontology Consortium website (http://www.geneontology.org), according to the predicted molecular functions, biological processes, and cellular components. Kyoto Encyclopedia of Genes and Genomes (KEGG) analysis was done using the routine settings (http://www.genome.jp/kegg/). The predicted interactions between differentially expressed lncRNAs and mRNAs were further analyzed based on their specific expression levels by construction of the Coding–Non-Coding (CNC) Network using the Cytoscape software.

### 2.5 Cell culture and transfection

SK-N-SH cells were obtained from the American Type Culture Collection (ATCC, Manassas, VA, USA) and cultured in Dulbecco’s modified Eagle’s medium (Thermo Fisher Scientific, Waltham, MA, USA) containing 10% fetal bovine serum (FBS; Thermo Fisher Scientific) and 4 mM glutamine (Thermo Fisher Scientific) at 37°C in a humidified atmosphere supplied with 5% CO_2_. The suppression of ASXL3 gene expression was realized by transfecting cells with ASXL3-siRNAs or the corresponding siRNA negative control using the Lipofectamine™ RNAiMAX Transfection Reagent (Invitrogen, Carlsbad, CA, USA) following the manufacturer’s instructions. The ASXL3 siRNAs, including ASXL3-Homo-2743, ASXL3-Homo-3617, and ASXL3-Homo-5299, were purchased from the GenePharma (Shanghai, China) and are listed in Table 2. The ASXL3 gene expression was evaluated by qRT-PCR 48 h after the transfection.

**Table 2.** ASXL3 siRNA sequences used for cell transfection

siRNA name	Sense sequences 1 (5'-3')	Antisense sequences 2 (5'-3')
ASXL3-Homo-2743	GCUUCCAUCUGCUAAAUUATT	UAAUUUAGCAGAUGGAAGCTT
ASXL3-Homo-3617	CCAAGUAAACUUCCAGAAATT	UUUCUGGAAGUUUACUUGGTT
ASXL3-Homo-5299	GCUGGUGACGCAGUUACUATT	UAGUAACUGCGUCACCAGCTT

### 2.6 Cellular function analysis

We performed a 3-(4,5-dimethylthiazol-2-yl)-5-(3-carboxymethoxyphenyl)-2-(4-sulfophenyl)-2 H-tetrazolium (MTS) assay and a flow cytometry apoptosis assay as described in our previous study [22]. Cell cycle progression of cultured cells were analyzed by flow cytometry using Tali™ Cell Cycle Kit (Thermo Fisher Scientific). After incubation with Cell Cycle solution for 25 min in the dark, the percentage of cells at the different cell cycle stages were determined using a flow cytometer (BD Biosciences, San Jose, CA, USA).

### 2.7 Immunofluorescence detection

The expression of Class III beta-tubulin isotype (betaIII-tubulin) in cultured cells was assessed using the FITC Immunofluorescence Detection Kit (Sangon Biology, Shanghai, China) following the manufacturer’s instructions. Briefly, cell slides were incubated with the blocking solution for 30 min at room temperature and with the primary antibody (Anti-beta III Tubulin antibody, #ab25770, Abcam, Cambridge, MA, USA) for 1 h. After four washes with phosphate-buffered saline containing Tween 20, the slides were incubated with the fluorescent-labeled secondary antibody and the nuclei were stained with DAPI. The slides were observed under a fluorescence microscope XSP-63B (Shanghai optical instrument factury, Shanghai, China).

### 2.8 Western blotting

Western blotting was performed as described in our previous study [22].

### 2.9 Statistical analyses

Data from at least three biological replicates are presented as mean ± standard deviation. The significance of the differences was evaluated by Student T-test (for two groups) or analysis of variance (for more than three groups) using the SPSS 18.0 software. The differences were considered significant for a P-value < 0.05.

## 3. Results

Significant alterations of the lncRNA and mRNA expression profiles in mouse myocardial tissues are caused by compound mutations of the ASXL3 gene. Therefore, we speculated that compound mutations of the ASXL3 gene induced changes in the lncRNA and mRNA expression profiles in the nervous system. In this study, we showed that the expression levels of many lncRNAs and mRNAs were changed in the cerebrum and cerebellum of mice with ASXL3 mutations. ASXL3 silencing inhibited neural cell proliferation and promoted cell apoptosis. These finding provide novel insights into the roles of the ASXL3 gene and lncRNA-mRNA interactions in brain functions and neurological disorders.

### 3.1 ASLX3 gene expression in the cerebrum and cerebellum of double mutant mice

**Figure 1.**
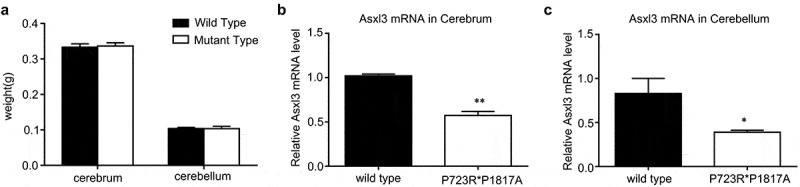
**Weight and ASL3 expression analyses in the mouse cerebrum and cerebellum**. (a) Weight of the cerebrum and cerebellum of wild-type C57BL/6 control and ASXL3 P723R*P1817A mutant mice. (b) ASXL3 mRNA levels in the cerebrum of wild-type and ASXL3 P723R*P1817A mutant mice. (c) ASXL3 mRNA expression in the cerebellum of wild-type and ASXL3 P723R*P1817A mutant mice. ASXL3 expression was evaluated by qRT-PCR. ASXL3: additional sex combs-like 3; qRT-PCR: quantitative real-time polymerase chain reaction; mRNA: messenger RNA; * and ** indicate P < 0.05 and P < 0.01, respectively

To analyze the effect of ASXL3 compound mutations on lncRNA and mRNA expression profiles in the cerebrum and cerebellum, ASXL3 double mutant mice (P723R*P1817A) were established as mentioned above. The weights of the mouse cerebrum and cerebellum were measured immediately after tissue collection. There was no significant change in the weights of the cerebrum and cerebellum between double mutant and wild-type C57BL/6 mice (Figure 1a). Subsequently, the expression of the ASXL3 gene in cerebrum and cerebellum tissues was determined by qRT-PCR. The ASXL3 mRNA levels in the cerebrum and cerebellum of ASXL3 P723R*P1817A mutant mice were significantly decreased compared with those in the wild-type mice (Figure 1b and 1c). These mouse cerebrum and cerebellum tissues were used for the following lncRNA and mRNA profile analysis.

### 3.2 Alteration of the lncRNA and mRNA expression profiles in the mouse cerebrum

**Figure 2.**
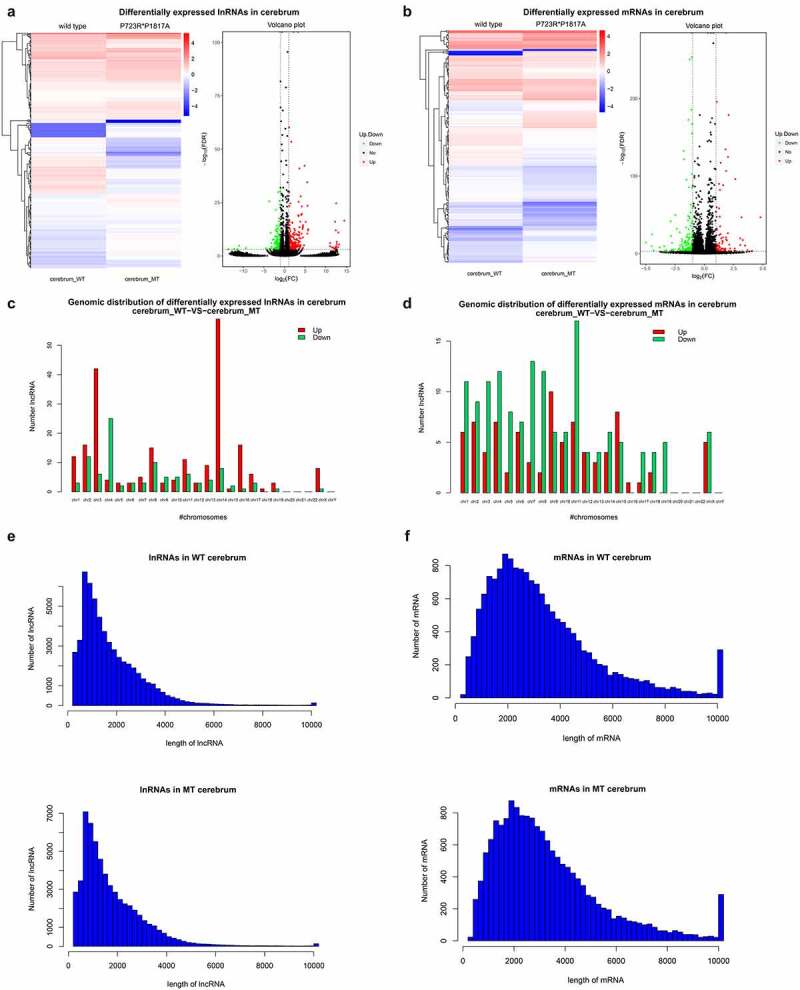
**Differential expression of lncRNAs and mRNAs in the ASXL3 mutant mouse cerebrum**. (a, b) Differential expression of lncRNAs (a) and mRNAs (b) in the cerebrum of wild-type and ASXL3 P723R*P1817A mutant mice. The differential expression of lncRNAs and mRNAs are presented as heatmap (left) and volcano plot (right). (c, d) Genomic distributions of lncRNAs (c) and mRNAs (d) differentially expressed in the cerebrum of wild-type and ASXL3 P723R*P1817A mutant mice. Upregulated and downregulated transcripts are shown as red and green bars, respectively. (e, f) Lengths of lncRNAs (e) and mRNAs (f) differentially expressed in the cerebrum of wild-type and ASXL3 P723R*P1817A mutant mice. ASXL3: additional sex combs-like 3; WT: wild-type; MT: mutant; mRNAs: messenger RNAs; lncRNAs: long non-coding RNAs

To explore the effects of ASXL3 P723R*P1817A mutations on the expression of genes and lncRNAs, we used RNA-seq to compare lncRNA and mRNA expression profiles in the cerebrum of wild-type and ASXL3 P723R*P1817A mutant mice. We identified 319 lncRNAs differentially expressed between the wild-type and mutant mice. Specifically, 212 lncRNAs were upregulated and 107 were downregulated in mutant mice compared to the levels in wild-type animals (Figure 2a and Supplemental Table 1). Additionally, 252 mRNAs, 91 upregulated and 161 downregulated, were differentially expressed in the cerebrum of ASXL3 P723R*P1817A mutant mice compared with their expression in wild-type mice (Figure 2b and Supplemental Table 2). The great number of lncRNAs and mRNAs affected by ASXL3 compound point mutations in the mouse cerebrum suggests a multifaceted role of the ASXL3 gene during cerebrum development and functioning.

Furthermore, the differentially expressed lncRNAs in the cerebrum of mutant mice were encoded by genomic sequences distributed in most mouse chromosomes (Figure 2c). Upregulated lncRNAs were highly enriched in Chromosomes 3 and 14, while downregulated lncRNAs were significantly present in Chromosome 4 (Figure 2c). Differentially expressed mRNAs in mouse cerebrum were also widely distributed on mouse chromosomes, most of them being encoded by Chromosome 11 (Figure 2d). The lncRNA lengths were variable, with most lncRNAs being shorter than 2000 bp (Figure 2e). Similarly, the lengths of mRNAs in the cerebrum were also variable, with most mRNAs were shorter than 6000 bp (figure 2f).

### 3.3 Functional categorization of differentially expressed mRNAs in the mouse cerebrum

**Figure 3.**
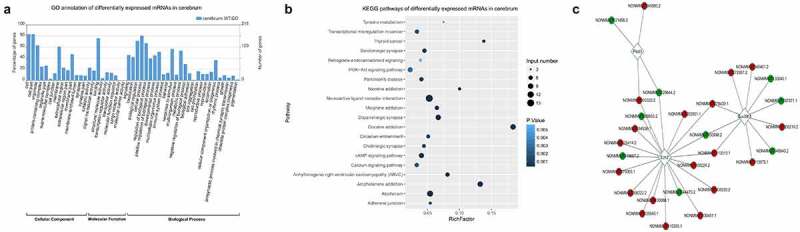
**Functional annotation of mRNAs differentially expressed in the ASXL3 mutant mouse cerebrum**. (a) GO categorization of mRNAs differentially expressed in the cerebrum of ASXL3 P723R*P1817A mutant mice. The mRNAs were categorized according to their annotated subcellular components, molecular functions, and biological processes. (b) Signaling pathways associated with the mRNAs differentially expressed in the cerebrum of ASXL3 P723R*P1817A mutant mice. These signaling pathways were analyzed by the KEGG method. (c) Coding-Non-Coding network based on the co-expression of lncRNAs and mRNAs differentially expressed in the cerebrum of mice with ASXL3 P723R*P1817A mutations. The network was constructed using upregulated (red) and downregulated (green) lncRNAs in the mutant mouse cerebrum. Plod3: 2-oxoglutarate 5-dioxygenase 3; EZH2: enhancer of zeste human homolog 2; SUV39H1: suppressor of variegation 3–9 homolog 1; KEGG: Kyoto encyclopedia of genes and genomes; GO: gene ontology; mRNAs: messenger RNAs

To obtain more functional information regarding the effects of ASLX3 mutations, the mRNAs differentially expressed in the cerebrum of wild-type and mutant mice were further analyzed by bioinformatics. These mRNAs were associated with multiple cellular components including protein-containing complexes, supermolecular complexes, cell junctions, membranes, extracellular regions, membrane-enclosed lumen, and synapses (Figure 3a). The differentially expressed mRNAs possessed various molecular activities including catalytic, signaling transduction, binding, structural, transcriptional regulating, transporter, cargo receptor, and molecular carrier activities (Figure 3a). In addition, the mRNAs differentially expressed in the mouse cerebrum were involved in several biological processes such as growth, development, immune system, responses to stimulus, metabolic processes, biological adhesion, cell aggregation, proliferation, reproduction, locomotion, rhythmic process, and pigmentation (Figure 3a). The large number of functional categories containing the differentially expressed mRNAs suggested the prevalent involvement of the ASXL3 gene in physiological and pathogenic processes in cerebrum.

KEGG annotation revealed that mRNAs differentially expressed in the mutant mouse cerebrum were enriched in a number of signaling pathways including tyrosine metabolism, transcriptional mis-regulation in cancer, thyroid cancer, serotonergic synapse, retrograde endocannabinoid signaling, PI3K-Akt signaling, Parkinson’s disease, nicotine addiction, neuro-active ligand-receptor interaction, morphine addiction, dopaminergic synapse, cocaine addiction, circadian entrainment, cholinergic synapse, cAMP signaling, calcium signaling, amphetamine addition, and alcoholism (Figure 3b). By constructing CNC Network based on the co-expression of lncRNAs and mRNAs, we found that the expression of 2-oxoglutarate 5-dioxygenase 3 (Plod3), enhancer of zeste human homolog 2 (EZH2), and suppressor of variegation 3–9 homolog 1 (SUV39H1), as well as many of their interacting lncRNAs, were significantly affected by the ASXL3 P723R*P1817A mutations (Figure 3c).

### 3.4 Differential expression of lncRNAs and mRNAs in the mouse cerebellum

**Figure 4.**
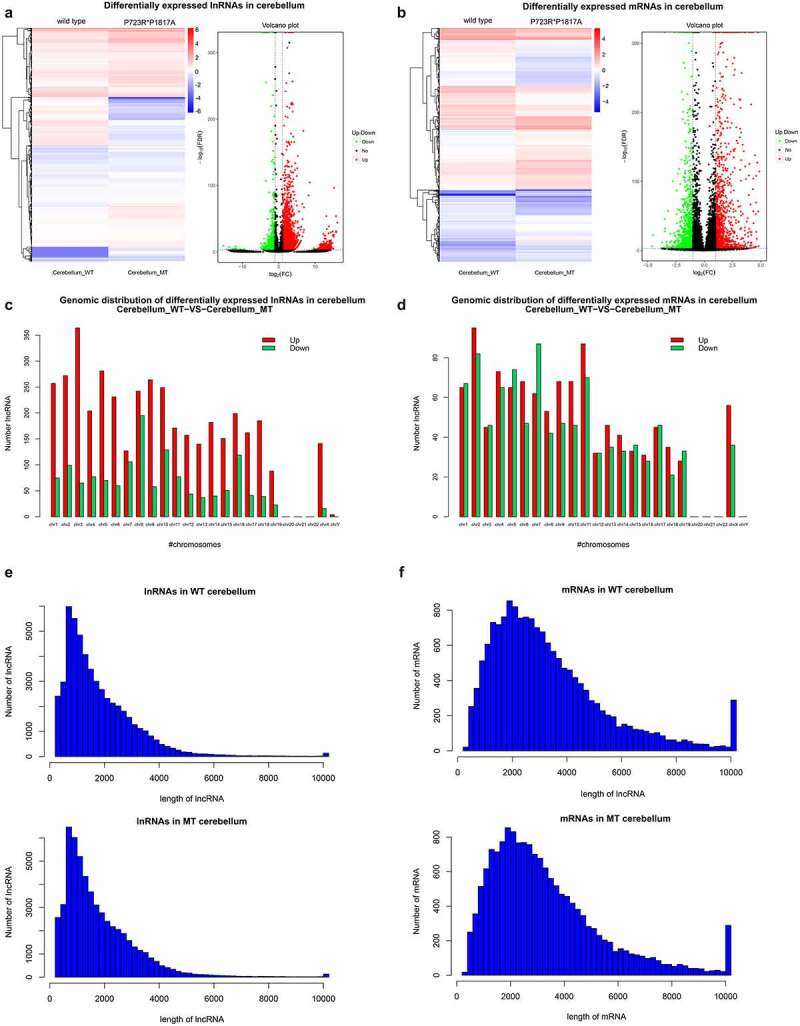
**Differential expression of lncRNAs and mRNAs in the cerebellum of ASXL3 mutant mice**. (a, b) Differential expression of lncRNAs (a) and mRNAs (b) in the cerebellum of wild-type and ASXL3 P723R*P1817A mutant mice. The expression of lncRNAs and mRNAs in mutant mice was compared with that of wild-type mice and are represented as heatmap (left) and volcano plot (right). (c, d) Chromosomal distribution of differentially expressed lncRNAs (c) and mRNAs (d) in the cerebellum of wild-type and ASXL3 P723R*P1817A mutant mice. Upregulated and downregulated transcripts are indicated by red and green bars, respectively. (e, f) Statistical analysis of the length of differentially expressed lncRNAs (e) and mRNAs (f) in the cerebellum of wild-type and ASXL3 P723R*P1817A mutant mice. ASXL3: additional sex combs-like 3; WT: wild-type; MT: mutant; mRNAs: messenger RNAs; lncRNAs: long non-coding RNAs

To study the influence of ASXL3 P723R*P1817A mutations on cerebellum genes and lncRNAs expression, lncRNA and mRNA expression profiles in the cerebellum of wild-type and ASXL3 P723R*P1817A mutant mice were analyzed by RNA-seq. We detected 5330 lncRNAs differentially expressed in the cerebellum of wild-type and mutant mice, with 3891 upregulated and 1439 downregulated lncRNAs in ASXL3 mutant mice compared with the levels in wild-type mice (Figure 4a and Supplemental Table 3). Moreover, compared with those in wild-type mice, 2204 mRNAs were differentially expressed in the cerebellum of the ASXL3 P723R*P1817A mutant mice. Among those, 1180 mRNAs were significantly upregulated and 1024 mRNAs were downregulated (Figure 4b and Supplemental Table 4). These remarkable changes in lncRNA and mRNA expression profiles suggested an essential function of the ASXL3 gene in cerebellum biology and pathogenesis.

In addition, our genomic distribution analysis revealed that the lncRNAs differentially expressed in the mouse cerebellum were encoded on various mouse chromosomes (Figure 4c). Upregulated lncRNAs were mainly distributed in Chromosome 3 and downregulated lncRNAs were greatly enriched in Chromosome 8 (Figure 4c). On each chromosome there were significantly more lncRNAs upregulated than downregulated ones (Figure 4c). Similarly, mRNAs differentially expressed in the mutant mouse cerebellum were distributed across most mouse chromosomes, especially the Chromosome 2, which harbored the largest number of upregulated mRNAs and the second larger numbers of downregulated mRNAs (Figure 4d). The analysis of lncRNAs and mRNAs lengths in the mouse cerebellum provided similar results to those obtained in ASXL3 P723R*P1817A mutant mouse cerebrum (Figure 4e and 4f).

### 3.5 Functional categorization of mRNAs differentially expressed in the mouse cerebellum

**Figure 5.**
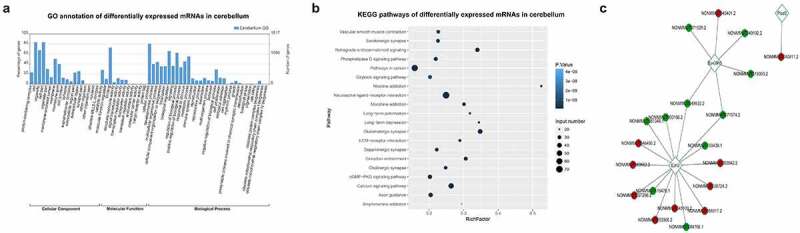
**Functional annotation of mRNAs differentially expressed in ASXL3 mutant mouse cerebellum**. (a) GO annotation of mRNAs differentially expressed in the cerebellum of mice with ASXL3 P723R*P1817A mutations. Differential mRNAs were categorized according to their subcellular components, molecular functions, and biological processes. (b) KEGG pathways of mRNAs differentially expressed in the cerebellum of ASXL3 P723R*P1817A mutant mice. KEGG signaling pathways with a significant enrichment of the differentially expressed mRNAs are shown. (c) Coding-Non-Coding network of lncRNAs and mRNAs differentially expressed in the cerebellum of ASXL3 P723R*P1817A mutant mice. LncRNAs upregulated (red) and downregulated (green) in the mutant mouse cerebellum were used for network construction. Plod3: 2-oxoglutarate 5-dioxygenase 3; EZH2: enhancer of zeste human homolog 2; SUV39H1: suppressor of variegation 3–9 homolog 1; KEGG: Kyoto encyclopedia of genes and genomes; GO: gene ontology; mRNAs: messenger RNAs

To provide insights into the effects of ASLX3 mutations in the cerebellum, mRNAs differentially expressed between the wild-type and mutant mouse cerebellum tissues were subsequently categorized by bioinformatics. The GO analysis revealed that mRNAs differentially expressed in the cerebellum belonged to many cellular components including membrane-enclosed lumen, cell junction, synapse, supramolecular complex, extracellular region, and nucleoid (Figure 5a). Regarding the molecular functions, mRNAs differentially expressed in the mouse cerebellum were greatly enriched in catalytic, molecular function regulator, binding, structural molecule, signaling transducer, transcription regulator, transporter, translation regulator, antioxidant, molecular carrier, and cargo receptor activities (Figure 5a). Additionally, the mRNA differentially expressed in the cerebellum were linked with various biological processes including development, cellular component organization, biogenesis, localization, signaling, responses to stimulus, metabolism, immune system, reproduction, locomotion, behavior, cell proliferation and aggregation, rhythmic process, growth, chemical synaptic transmission, detoxification, pigmentation, cell killing, and protein complex biogenesis (Figure 5a).

We also showed by KEGG analysis that mRNAs differentially expressed in the mutant mouse cerebellum were significantly associated with multiple biological pathways, including vascular smooth muscle contraction, serotonergic synapse, retrograde endocannabinoid signaling, phospholipase D signaling, pathways in cancer, oxytocin signaling, nicotine addiction, neuro-active ligand–receptor interaction, morphine addiction, long-term potentiation, long-term depression, glutamatergic synapse, ECM–receptor interaction, dopaminergic synapse, circadian entrainment, cholinergic synapse, cGMP-PKG signaling, calcium signaling and axon guidance, and amphetamine addiction (Figure 5b). The construction of a CNC Network also showed that the differential expression of Plod3, EZH2, and SUV39H1 induced by ASXL3 mutations correlated with their interacting lncRNAs (Figure 5c).

### 3.6 Validation of lncRNA and mRNA expression profiles in the mouse cerebrum and cerebellum

**Figure 6.**
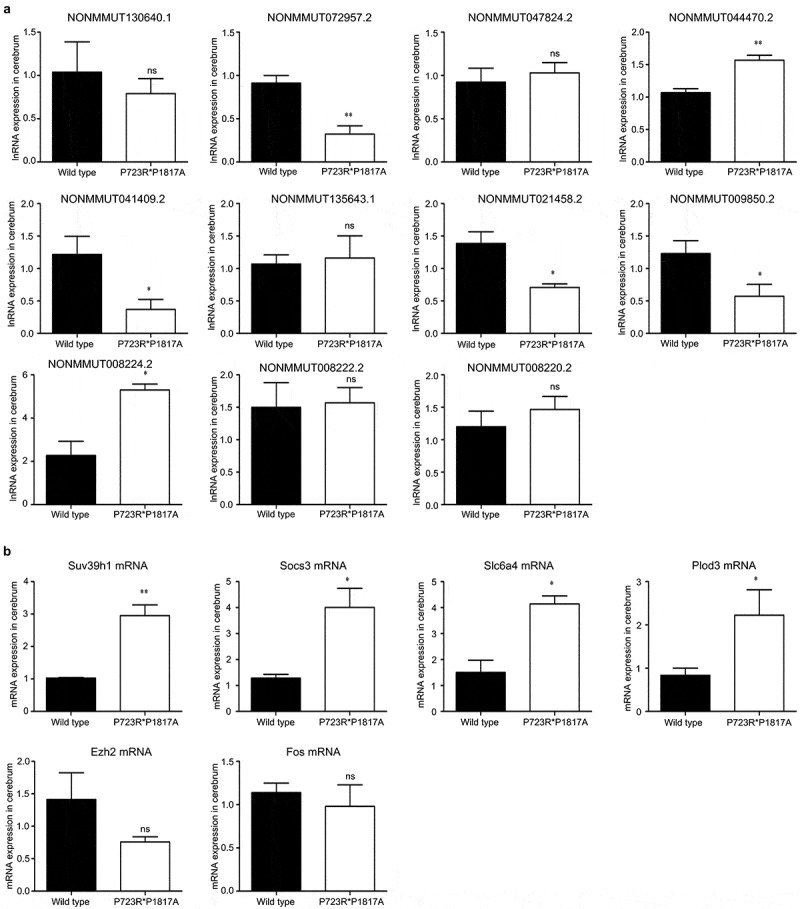
**Validation of lncRNA and mRNA expression levels in the ASXL3 mutant mouse cerebrum**. (a) Relative expression of eleven lncRNAs in the cerebrum of wild-type and ASXL3 P723R*P1817A mutant mice. We analyzed the LncRNAs levels by qRT-PCR. (b) Relative expression of six functional genes in the cerebrum of wild-type and ASXL3 P723R*P1817A mutant mice. A qRT-PCR analysis was performed to compare the mRNA levels. Plod3: 2-oxoglutarate 5-dioxygenase 3; EZH2: enhancer of zeste human homolog 2; SUV39H1: suppressor of variegation 3–9 homolog 1; SOCS3: suppressor of cytokine signaling 3; Slc6a4: serotonin transporter gene; qRT-PCR: quantitative real-time polymerase chain reaction; ns: no significant; mRNAs: messenger RNAs; lncRNAs: long non-coding RNAs; * and ** indicate P < 0.05 and P < 0.01, respectively

To validate the lncRNA and mRNA expression profiles detected by RNA-seq, eleven lncRNAs differentially expressed in the mouse cerebrum were first confirmed by qRT-PCR. The levels of four lncRNAs were significantly decreased in the cerebrum of ASXL3 mutant mice, whereas the expression of two other lncRNAs was remarkably increased in the mutant mouse cerebrum (Figure 6a). In addition, the mRNA levels of four functional genes, namely, SUV39H1, suppressor of cytokine signaling 3 (SOCS3), serotonin transporter gene Slc6a4, and Plod3, were also confirmed to be greatly elevated in the cerebrum of mice with P723R*P1817A mutations in the ASXL3 gene (Figure 6b).

**Figure 7.**
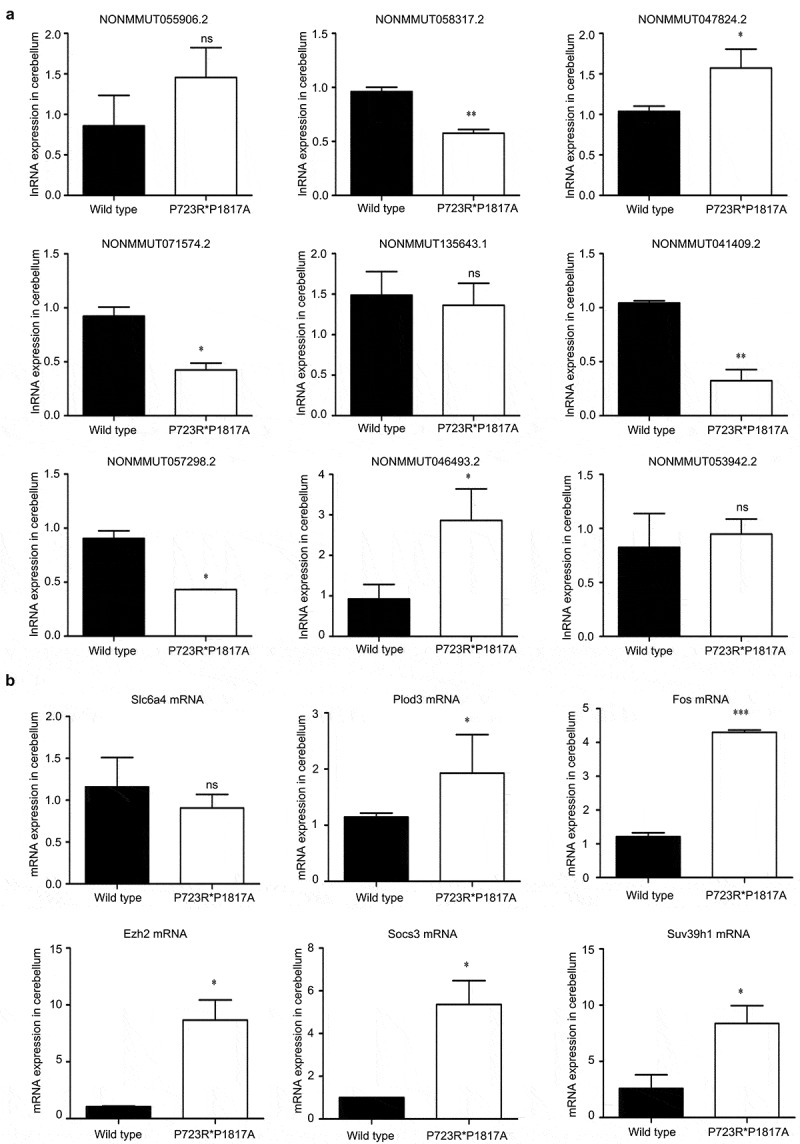
**Validation of lncRNA and mRNA expression levels in the ASXL3 mutant mouse cerebellum**. (a) Relative expression levels of nine lncRNAs in the cerebellum of wild-type and ASXL3 P723R*P1817A mutant mice. We analyzed the lncRNAs levels by qRT-PCR. (b) Relative mRNA expression levels of six genes in the cerebellum of wild-type and ASXL3 P723R*P1817A mutant mice. The mRNA levels were determined by qRT-PCR. Slc6a4: serotonin transporter gene; Plod3: 2-oxoglutarate 5-dioxygenase 3; EZH2: enhancer of zeste human homolog 2; SOCS3: suppressor of cytokine signaling 3; SUV39H1: suppressor of variegation 3–9 homolog 1; qRT-PCR: quantitative real-time polymerase chain reaction; ns: no significant; mRNAs: messenger RNAs; lncRNAs: long non-coding RNAs; *, **, and *** indicate P < 0.05, P < 0.01, and P < 0.001, respectively

The expression levels of nine lncRNAs differentially expressed in the cerebellum of mutant mice were also confirmed by qRT-PCR. The expression of four of these lncRNAs was significantly reduced in the cerebellum of ASXL2 mutant mice compared with that in wild-type mice (Figure 7a). The expression of two other lncRNAs was remarkably enhanced in the cerebellum of mutant mice compared with wild-type levels (Figure 7a). The mRNA levels of five functional genes, Plod3, Fos, Ezh2, SOCS3, and SUV39H1, in the cerebellum of ASXL3 P723R*P1817A mutant mice were significantly greater than those in the wild-type animals (Figure 7b).

### 3.7 Modulation of neural cell functions by ASXL3 knockdown

**Figure 8.**
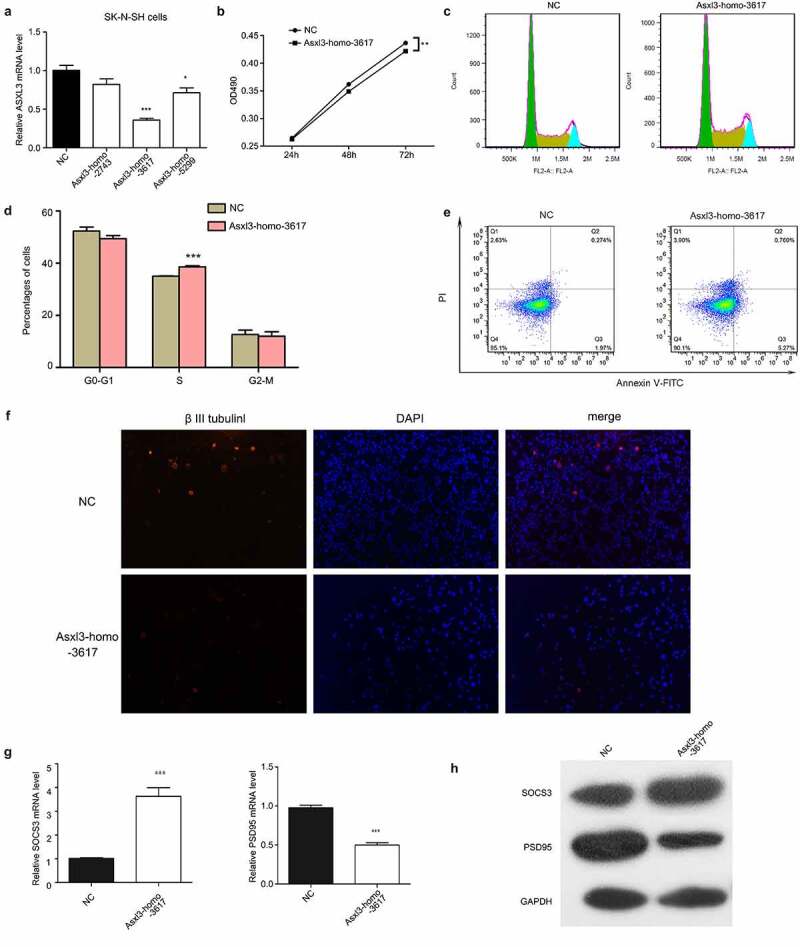
**Neural cell functions affected by siRNA-mediated ASXL3 depletion**. (a) Relative expression levels of ASXL3 in SK-N-SH neuroblastoma cells transfected with ASXL3 siRNAs was assessed by qRT-PCR. (b) Proliferation rates of SK-N-SH neuroblastoma cells transfected with ASXL3 siRNAs was measured using the MTS method. (c, d) Cell cycle progression of SK-N-SH neuroblastoma cells transfected with ASXL3 siRNAs determined by flow cytometry. (e) Apoptosis of SK-N-SH neuroblastoma cells transfected with ASXL3 siRNAs evaluated by flow cytometry. (f) Immunofluorescence analysis of the expression of betaIII-tubulin in SK-N-SH neuroblastoma cells. (g) Relative mRNA levels of Socs3 and PSD96 in SK-N-SH neuroblastoma cells was determined by qRT-PCR. (h) Western blot analysis of Socs3 and PSD96 protein abundance in SK-N-SH neuroblastoma cells. NC: negative control; ASXL3: additional sex combs-like 3; betaIII-tubulin: Class III beta-tubulin isotype; PSD95: postsynaptic density-95; qRT-PCR: quantitative real-time polymerase chain reaction; MTS: 3-(4,5-dimethylthiazol-2-yl)-5-(3-carboxymethoxyphenyl)-2-(4- sulfophenyl)-2 H-tetrazolium; mRNAs: messenger RNAs; *, **, and *** indicate P < 0.05, P < 0.01, and P < 0.001, respectively

To obtain more insights into the biological roles of ASXL3, SK-N-SH neuroblastoma cells were transfected with siRNAs targeting the ASXL3 gene. The siRNAs, especially Asxl3-homo-3617, greatly downregulated ASXL3 expression (Figure 8a). The proliferation of SK-N-SH cells transfected with ASXL3 siRNAs was decreased compared with that of cells transfected with the negative control (Figure 8b). In addition, ASXL3 knockdown induced an increased number of SK-N-SH cells in S stage (Figure 8c and 8d). The apoptosis levels of SK-N-SH cells was also remarkably increased by the ASXL3 knockdown (Figure 8e). Furthermore, the suppression of ASXL3 gene expression in SK-N-SH cells caused a significant repression of betaIII-tubulin expression (figure 8f), promoted Socs3 expression, and inhibited postsynaptic density-95 (PSD95) expression (Figure 8g and 8h). These results revealed a significant effect of ASXL3 expression on neural cell functions, which is consistent with the significant changes in lncRNAs and mRNAs expression profiles induced by ASXL3 point mutations and the resultant decreased ASXL3 expression levels.

## 4. Discussion

It has been established that lncRNAs constitute a critical regulatory module in various processes associated with physiological functions and pathogenesis [23]. Large-scale transcriptome analysis based on RNA-seq is an efficient strategy for investigating lncRNA profiles because it allows instant identification and quantification of the expression of large amounts of lncRNAs [24]. Regarding the great influence of lncRNAs on gene expression [11], the simultaneous characterization of lncRNA and mRNA expression profiles by transcriptome sequencing has provided valuable insights into the mechanistic properties of key lncRNAs during several pathogenic processes [25,26]. Our previous study identified compound heterozygous mutations in the ASXL3 gene in a Chinese family presenting congenital heart disease. Further investigations using a mouse model showed that these ASXL3 mutations caused great alterations of the lncRNA and mRNA expression profiles in heart tissues [22], suggesting that ASXL3 mutations might be a critical event regulating lncRNAs and mRNAs expression. The ASXL3 gene has been previously involved in neural cell function and neurological processes [20,21]. However, little is known about lncRNAs and mRNAs expression regulation in brain tissues.

To provide a general view of the influence of ASXL3 compound mutations on lncRNAs and mRNAs expression in the brain, we generated an ASXL3 P723R*P1817A mutant mice, in which ASXL3 gene expression is greatly decreased. A transcriptome analysis based on the RNA-seq technique revealed that the expression of many lncRNAs and mRNAs were affected by the introduction of ASXL3 P723R*P1817A mutations. Moreover, these differentially expressed lncRNAs and mRNAs were widely distributed across almost all mouse chromosomes. We identified much more upregulated than downregulated lncRNAs in the ASXL3 mutant mouse cerebellum. This observation suggested that the upregulation of lncRNAs expression in the mouse cerebellum are an important molecular mechanism underlying cerebellum physiology and pathogenic processes.

The bioinformatics analysis revealed an enrichment of the differentially expressed lncRNAs and mRNAs in many cellular components and molecular functions. The great variety of lncRNAs and mRNAs affected by the ASXL3 gene mutations suggest that transcriptome alterations regulated by ASXL3 are essential for neural cell functions. For instance, synapses are critical intercellular structures passing electrical or chemical signals from one neuron to another or to an effector cell during neurotransmission, which is often disturbed in neurological disorders such as Alzheimer’s disease and spinal cord injury [27]. The lncRNA GM12371 was recently reported to regulate synapse morphology, synaptic communication, and synapse density by modulating the transcription of a group of genes associated with neuronal development and differentiation [28]. In the present study, we identified many lncRNAs distributed in synapses, including dopaminergic, cholinergic, serotonergic, and glutamatergic synapses, and involved in synapse-related biological processes. These observations indicate that ASXL3-regulated lncRNAs and mRNAs might be involved in the functioning of various synapse types. This deserves further investigations.

The KEGG annotation showed that numerous functional genes associated with many signaling pathways in the cerebrum and cerebellum were regulated by ASXL3 mutations. For instance, PI3K/AKT signaling is involved in the regulation of functions such as neuronal migration and apoptosis [29,30]. Moreover, the PI3K/AKT pathway was previously shown to be regulated by lncRNAs [31]. Our analysis further suggests that the interaction of lncRNA with the PI3K/AKT signaling pathway plays physiological and pathogenic roles in neuronal cells. The CNC Networks predicted the interaction of numerous differentially expressed lncRNAs with three functional genes, including EZH2, Plod3, and SUV39H1. The inhibition of EZH2 activity was shown to suppress bupivacaine-induced neuronal apoptosis in spinal cord dorsal root ganglia [32]. Overexpression of the PLOD3 gene, which encodes a lysyl hydroxylase, significantly promotes the progression of gliomas and is associated with a poor prognosis in glioma patients [33]. Suv39h1 is a methyltransferase that was characterized as a chromatin modifier regulating neuron survival, neuron death, and trauma progression [34]. However, the molecular mechanisms regulating the role of these three genes in neuron physiology and neurological disorders remains poorly understood. The identification of many lncRNAs influenced by the ASXL3 mutation in the mouse cerebrum and cerebellum suggests that lncRNA-mediated transcription regulation controls the expression of these genes in neural cells during neurological disorder progression.

Finally, our cellular assays based on ASXL3 gene silencing confirmed ASXL3 involvement in neural cell proliferation, cell cycle progression, and apoptosis. ASXL3 gene depletion greatly altered the expression of betaIII-tubulin, Socs3, and PSD96 genes, which are all closely linked to synapse formation and neuronal functions [35,36]. This further supports a potential role for ASXL3-regulated lncRNAs and mRNAs in neural cells and requires further investigations. Whether the alterations of lncRNA and mRNA expression profiles caused by the compound mutations in the ASXL3 gene are involved in other biological and pathogenic processes is also worth studying. For instance, previous reports showed that ASXL3 acts as a novel pluripotency factor in respiratory epithelial cells associated with small cell lung cancer [19]. Moreover, lncRNAs were recently linked with invasion and metastasis of small cell lung cancer [37,38]. Based on the significant changes in lncRNAs and mRNAs expression induced by ASXL3 mutations reported here, lncRNAs and their interacting mRNAs networks might constitute biomarkers, treatment targets, or critical molecular events mediating the pathogenesis of diseases. Their therapeutic potential might be exploited in a similar fashion than miRNA and fungi are used in the fight against cancer and COVID-19 [39–41].

This study is a preliminary exploration and additional work is needed to confirm our findings by overexpression or inhibition of lncRNAs or mRNAs in animal models.

## 5. Conclusion

In summary, many lncRNAs and mRNAs were differentially expressed in the cerebrum and cerebellum of an ASXL3 mutant mouse. Our work provides novel insights into the roles of the ASXL3 gene and lncRNA-mRNA interactions in brain functions and neurological disorders.

## Supplementary Material

Supplemental Material

## Data Availability

The datasets generated during and/or analyzed during the current study are available from the corresponding author on reasonable request.

## Highlights

1. The expression of lncRNAs and mRNAs is altered in ASXL3 mutant mouse cerebrum.
2. The expression of lncRNAs and mRNAs is also altered in ASXL3 mutant mouse cerebellum.
3. ASXL3 is involved in the proliferation, cell cycle, and apoptosis of neural cells
