# Supplementary material for: Alteration of long non-coding RNAs and mRNAs expression profiles by compound heterozygous ASXL3 mutations in the mouse brain
Source: Bioengineered. 2021 Sep 24;12(1):6935–51. doi: 10.1080/21655979.2021.1974811 (PMC8806560; doi:10.1080/21655979.2021.1974811)
Supplement: Supplemental Material [file KBIE_A_1974811_SM2258.zip › supplementary/Supplementary Materials.docx]

**Supplementary Materials**

**Supplemental Table 1. Differentially expressed lncRNAs in the cerebrum of wild-type and ASXL3 P723R*P1817A mutant mice.**

**Supplemental Table 2. Differentially expressed mRNAs in the cerebrum of wild-type and ASXL3 P723R*P1817A mutant mice.**

**Supplemental Table 3. Differentially expressed lncRNAs in the cerebellum of wild-type and ASXL3 P723R*P1817A mutant mice.**

**Supplemental Table 4. Differentially expressed mRNAs in the cerebellum of wild-type and ASXL3 P723R*P1817A mutant mice.**
